# Health care professionals overestimate the risk for locoregional recurrences after breast cancer treatment depending on their specialty

**DOI:** 10.1007/s10549-022-06549-9

**Published:** 2022-03-13

**Authors:** Jet W. Ankersmid, Pauline E. R. Spronk, Anneke M. Zeillemaker, Sabine Siesling

**Affiliations:** 1 Department of Health Technology and Services Research, Technical Medical Center, University of Twente, Enschede, The Netherlands; 2 Santeon Hospital Group, Utrecht, The Netherlands; 3 Department of Surgery, Alrijne Hospital, Leiden, The Netherlands; 4 Department of Research and Development, Netherlands Comprehensive Cancer Organisation, Utrecht, The Netherlands

**Keywords:** Breast cancer, Surveillance, Recurrence, Risk, Health care providers

## Abstract

**Purpose:**

For the implementation of personalised surveillance, it is important to create more awareness among HCPs with regard to the risk for locoregional recurrences (LRRs). The aim of this study is to evaluate the current awareness and estimations of individual risks for LRRs after completion of primary treatment for breast cancer among health care professionals (HCPs) in the Netherlands, without using any prediction tools.

**Methods:**

A cross-sectional survey was performed among 60 HCPs working in breast cancer care in seven Dutch hospitals and 25 general practitioners (GPs). The survey consisted of eleven realistic surgically treated breast cancer cases. HCPs were asked to estimate the 5-year risk for LRRs for each case, which was compared to the estimations by the INFLUENCE-nomogram using one-sample Wilcoxon tests. Differences in estimations between HCPs with different specialities were determined using Kruskal–Wallis tests and Dunn tests.

**Results:**

HCPs tended to structurally overestimate the 5-year risk for LRR on each case. Average overestimations ranged from 4.8 to 26.1%. Groups of HCPs with varying specialities differed significantly in risk estimations. GPs tended to overestimate the risk for LRRs on average the most (15.0%) and medical oncologists had the lowest average overestimation (2.7%).

**Conclusions:**

It is important to create more awareness of the risk for LRRs, which is a pre-requisite for the implementation of personalised surveillance after breast cancer. Besides education for HCPs, the use of prediction models such as the INFLUENCE-nomogram can support in estimating an objective estimate of each individual patient’s risk.

## Introduction

In the Netherlands, the incidence of breast cancer has been rising until 2019 and survival rates have improved [1], resulting in a growing number of breast cancer survivors eligible for follow-up care. Follow-up care can be subdivided into *aftercare* and *surveillance*. *Aftercare* focusses on informing on, monitoring and addressing of complaints, symptoms and (late) physical or psychosocial effects of the disease and treatment [2]. The aim of *surveillance* is early detection of locoregional recurrences (LRRs) or secondary primary breast tumours (SPs) [2]. Early detection of LRRs and SPs is important because it might prevent the development of subsequent distant metastasis and thereby improve survival after recurrence [3, 4]. Unlike the highly personalised breast cancer treatment, surveillance is predominantly ‘one-size-fits-all’: annual imaging (e.g. mammography and/or MRI) and physical examination for at least 5 year after treatment [2].

In general, patients treated for breast cancer have a low risk for LRRs. About 2.6% of Dutch women diagnosed with breast cancer between 2003 and 2006 developed a LRR in the first 5 years following primary treatment [5]. About half of all LRRs are detected by the women themselves in between routine surveillance visits [3, 5, 6]. In the detection of the other half of LRRs and SPs, breast imaging plays a major role and physical examination a minor role [3]. Furthermore, the risk for LRRs is not the same for every patient and is related to, i.e. patient, tumour, and treatment characteristics and changes over time [5], which confirms our knowledge on the biology of breast cancer as a heterogeneous disease. To lower the burden on health care (costs) and patients, the frequency and duration of surveillance should be adapted accordingly. For example, for patients with a low risk for LRR, surveillance could be less intensive in frequency and the duration could be shortened.

To identify patients with a low or high risk for LRRs who might benefit from less or more intensive surveillance, risk prediction models can be used. The INFLUENCE-nomogram [5] is an example of such a risk prediction model and is based on data of the Netherlands Cancer Registry (NCR). The INFLUENCE-nomogram was validated both internally (using bootstrapping) and externally (using NCR data) and has a satisfactory accuracy (c-statistic of 0.71 after validation) [5]. After entering patient, tumour, and treatment characteristics, it enables estimation of the individual risk for developing a LRR within the first five years after surgery, and the (conditional) annual risks. Whereas most risk prediction models are used to aid decision-making about treatment, the INFLUENCE-nomogram is aimed at predicting risks for LRRs after already received treatment and is therefore particularly suitable to aid decision-making about personalised post-treatment surveillance.

To support implementation of the INFLUENCE-nomogram and risk-based surveillance, it is important to evaluate to which extent health care professionals are currently aware of patients’ risk for LRR. The more the estimates of the health care professionals (HCPs) deviate from the objective risk estimate calculated using the INFLUENCE-nomogram, the more the nomogram can add value to decision-making regarding personalised surveillance. Moreover, existing and systematic deviations might form a barrier for the uptake of the INFLUENCE-nomogram. An objective risk estimate could also prevent HCPs from misinforming patients causing excessive fear of recurrence.

Therefore, the aim of this study is to evaluate the current awareness of and estimations of individual risks for LRR after finalising primary treatment for breast cancer among HCPs in the Netherlands and to compare these values with the risks estimated using the INFLUENCE-nomogram.

## Methods

In this study, a cross-sectional survey was performed among Health Care Professionals (HCPs) working in breast cancer care in seven hospitals (six teaching, one academic) in the western part of the Netherlands, which together form the “regional oncology network West” (RO-West). Moreover, GPs working within the same region were invited to participate.

### Participants and procedures

Per hospital, the chair of the Multidisciplinary Tumour Board (MDT), in most cases a breast cancer surgeon, was approached by one of the researchers involved in this study (PS) and was asked to participate in the study. Subsequently, this HCP made sure that the researcher could attend one of the MDT meetings. MDTs in the Netherlands consist of HCPs from all disciplines involved in breast cancer care, i.e. radiologists, pathologists, (plastic) surgical oncologists, medical oncologists, radiation oncologists, nurse practitioners and breast cancer nurses. At the start of the MDT meeting, the researcher explained the aim of the study, after which the attending HCPs in that particular meeting completed the survey individually. HCPs were not allowed to discuss anything while completing the survey. This approach was chosen, so that HCPs would be unprepared when answering the question. GPs were approached using a convenience sampling strategy. GPs (*n* = *35*) from the network of one of the researchers (AZ) were invited by e-mail. Participating GPs received the survey via e-mail.

### Materials

The survey was developed by the research team and was pre-tested with a sample of four HCPs in one hospital and was then modified based on the obtained feedback. The survey consisted of eleven realistic surgically treated breast cancer cases, each with different patient, tumour, and treatment characteristics. These characteristics included the following: the patient’s age; tumour characteristics (post-operative T-stage (pT); post-operative lymph node status (pN); receptor status (ER/PR); grade (Bloom -Richardson); and multifocality); and type of post-surgery treatment (radio-, anti-hormonal- and/or chemotherapy). Participants were asked to estimate the risk of LRR per case. Participants were not asked to estimate the risks for second primary tumours (SPs) because these could not be estimated by the INFLUENCE-nomogram version 1.0 and estimations could therefore not be compared to the nomogram’s estimation. Furthermore, of all cases, the risk of LRR was estimated using the INFLUENCE-nomogram (version 1.0) by filling in the variables in the nomogram on the Evidencio platform (https://www.evidencio.com/models/show/721). The cases that were presented to the HCPs are displayed in Table 1. The survey as presented to the HCPs can be found in Appendix I.

**Table 1:** Cases presented to HCPs

Case	Age	pT	pN	Tumour grade (Bloom-Richardson)	ER status	PR status	Multifocality	Radiotherapy	Anti- hormonal therapy	Chemotherapy
1a	53	T1	N0	2	+	+	No	X	X	
1b	53	T1	N0	2	+	+	No	X		
2	42	T2	N1	3	−	−	No	X		X
3a	63	T1c	N0	1	+	+	No	X		
3b	63	T1c	N0	1	+	+	No			
4a	70	T2	N1	2	+	−	Yes	X	X	X
4b	70	T2	N1	2	+	−	Yes	X	X	
5a	50	T3	N2	2	+	−	Yes	X	X	X
5b	50	T3	N2	2	+	−	Yes	X	X	
6a	74	T1b	N0	2	+	+	No	X	X	
6b	74	T1b	N0	2	+	+	No			

### Statistical analysis

Frequencies and percentages were used to display responses to individual questions. One-sample Wilcoxon tests were used to test whether the medians of the risks estimated by the HCPs were significantly different from the risk estimated using the INFLUENCE-nomogram. One-sample Wilcoxon tests were used instead of one-sample *T*-tests due to non-normal distributions of the data. To identify whether groups of HCPs with differing specialties differed on their risk estimations on the cases, Kruskal–Wallis tests were performed. For the cases on which the Kruskal–Wallis tests was significant, the Dunn test was performed as a post hoc analysis to determine which groups differed from each other. The Bonferroni correction was used to adjust p-values to control the family-wise error rate (FWER). All statistical analyses were performed in R version 4.1.0 (R Foundation for Statistical Computing, Vienna, Austria).

## Results

### Respondents

In total, 85 HCPs participated in the study, from which 60 (70.6%) were hospital HCPs and 25 (29.4%) were GPs. Most of the hospital HCPs were residents at the surgery department (*N* = *13*, 15.3%), nurse practitioners (*N* = *10*, 11.8%), or surgical oncologists (*N* = *10*, 11.8%) (Table 2). The number of participating HCPs varied per hospital from 19 (22.4%) to 3 (3.5%) participants.

**Table 2:** Respondent characteristics

Type of HCP	*N*	%
General practitioner	25	29.4
Resident surgery department	13	15.3
Surgical oncologist	10	11.8
Nurse practitioner	10	11.8
Radiologist	9	10.6
Radiotherapist	9	10.6
Medical oncologist	5	5.9
Breast cancer nurse	4	4.7
Total	85	

### Risk perceptions

In Table 3, the estimated 5-year risk per case by the HCPs is displayed together with the risk estimated using the INFLUENCE-nomogram. HCPs tended to overestimate the 5-year risk for LRR on each case that was presented to them. Wilcoxon signed rank tests revealed that in all cases, the median risk estimations by the HCPs were significantly higher than the risk estimation by the INFLUENCE-nomogram (all *p*-levels were < 0.001).

**Table 3:** Descriptive statistics of estimated five-year risks by the HCPs and risk estimation INFLUENCE-nomogram

Case	Risk estimation INFLUENCE-nomogram (%)^a^	N	Min	Max	Median	Mean	Std. deviation	Variance	Average overestimation (%)
1a	1.0	85	0.0	30	5^b^	5.80	5.30	28.04	4.8
1b	2.3	85	0.0	70	8^b^	10.64	10.07	101.31	8.4
2	7.2	85	3.0	60	15^b^	17.67	12.16	147.84	10.5
3a	1.2	85	0.0	30	5^b^	6.95	6.80	46.23	5.8
3b	2.3	85	0.1	70	10^b^	12.53	10.70	114.48	10.3
4a	0.7	85	1.0	50	10^b^	12.59	8.37	69.98	11.9
4b	1.7	85	0.5	80	19^b^	19.06	12.86	165.44	17.3
5a	1.6	85	1.5	60	20^b^	20.57	13.38	179.04	19.0
5b	3.6	84	2.5	90	25^b^	29.68	18.70	349.78	26.1
6a	0.6	63	0.0	30	5^b^	7.21	6.11	37.31	6.6
6b	2.9	62	0.1	40	10^b^	11.90	9.18	84.27	9.0

Results of Wilcoxon one-sample signed rank test

^a^LRR = locoregional recurrence. Estimated using the INFLUENCE-nomogram[7]: https://www.evidencio.com/models/show/721

^b^Median is significantly higher than risk estimation using the INFLUENCE-nomogram at the *p* < 0.001 level

**Fig. 1:**
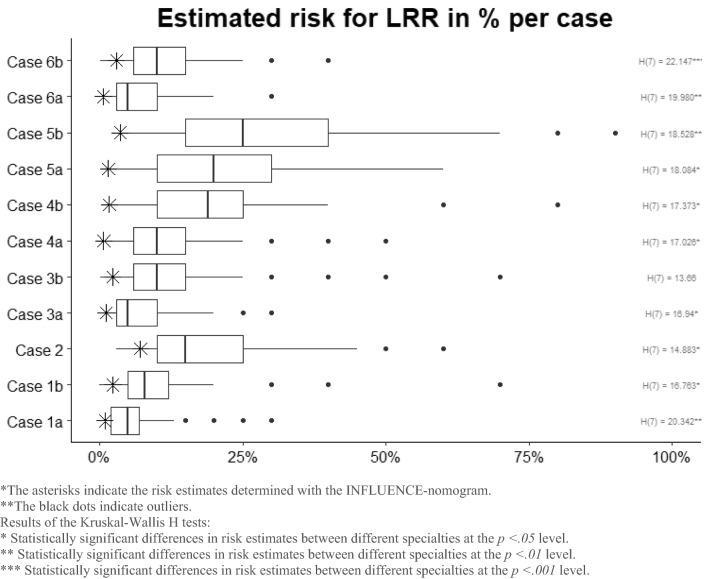
Estimated 5-year risks for locoregional recurrences on all cases

Average overestimations ranged from 4.84% on case 1a to 26.08% on case 5b (see Fig. 1). The highest variance on risk estimates was seen on cases 5b and 5a. The lowest variance was seen on cases 1a and 6a.

### Differences per specialty

**Fig. 2:**
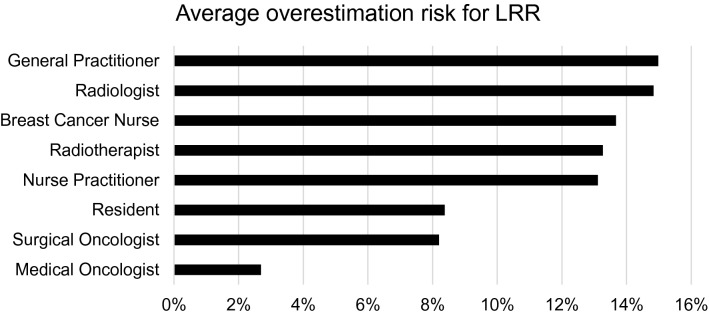
Average overestimations for all cases per specialty

In the table in Appendix I, the estimated 5-year risk per case is displayed per specialty of the HCPs. In Fig. 2, the average overestimation per discipline on all cases is displayed. GPs tended to overestimate the risk for LRRs the most (15.0%) and medical oncologists had the lowest average overestimation (2.7%).

Kruskal–Wallis *H* tests showed that there were statistically significant differences in the estimated risks between groups of HCPs with differing specialties on all cases except for case 3b (see Fig. 1 for statistical values per case).

Comparisons between all pairs of groups of HCPs revealed that for case 1b, case 4a, case 4b, case 5a, case 5b, case 6a, and case 6b, the median risk estimate of the GPs was significantly higher than the estimate by the medical oncologists. For case 5a and 5b, radiotherapists estimated the risks for LRR significantly higher than medical oncologists. Furthermore, for case 1a median risk estimates differed significantly for breast cancer nurses and medical oncologists; breast cancer nurses and residents; and general practitioners and residents. Nurse practitioners estimated the risk on case 6a higher than medical oncologists.

## Discussion

In this study, we evaluated the current awareness and estimations of risks for LRRs after breast cancer among HCPs in the Netherlands, without using prediction tools. We found that HCPs structurally overestimate the risk for LRRs. The average overestimations on the cases ranged from about 4% to 26%. Furthermore, HCPs with varying specialties differed significantly in risk estimations; GPs tended to overestimate the risk for LRRs the most (15.0%) and medical oncologists had the lowest average overestimation (2.7%).

Variations in the risk estimations were smallest on the case of a 53-year-old women with a hormone positive small tumour (< 1 cm) without lymph node involvement and treated with radiation and anti-hormonal therapy (case 1a). Case 1a can be seen as a common case in terms of tumour characteristics. Variations were largest on case 5b, that of a 50-year-old woman with a ER positive, PR negative big tumour (> 5 cm) with lymph node involvement and multifocality which was treated with radio- and anti-hormonal therapy, but without chemotherapy. Case 5b is obviously harder to predict for HCPs. An explanation for this could be that in earlier years of research, tumour size in combination with the extent of lymph node involvement have always been the most important prognostic factors for breast cancer. However, from more recent research, we know that tumour biology (molecular signature and grading) is more decisive. Moreover, targeted therapies may even decrease the risk of LRRs in initially advanced staged breast cancer when a pathologic complete response is achieved [7].

That medical oncologists have more knowledge about the effect of targeted therapies on the risk of LRRs, is clearly demonstrated by our study. There was a significant difference in risk estimates between groups of HCPs with differing specialties and medical oncologists tended to estimate the risk for LRRs the most accurately. Another explanation for the more accurate estimates could be the fact that medical oncologists have more experience with the use of risk information and prediction models (such as Predict [8]) in decision-making regarding adjuvant systemic therapy. Zikmund-Fisher et al. (2016) found, for example, that medical oncologists were way more likely to quantify risk estimates for patients than surgical oncologists [9].

The large overestimation by GPs could be due to a knowledge-gap on the effects of different therapies, experience with estimating risks for LRR due to a small number of patients with breast cancer in their practice, and the use of prediction models. GPs are currently hardly actively involved in surveillance after breast cancer [10]. However, this may change as follow-up shifts more towards a patient-led flexible model. This may lead to more involvement of the GP in surveillance. It is therefore important that GPs are included in education about risk-based surveillance after breast cancer and on how to use the INFLUENCE-nomogram to calculate objective risk estimates. Furthermore, GPs need to be educated about the patient-, tumour-, and treatment-related factors that influence disease recurrence.

In this study, we see that the risks for LRRs estimated by HCPs deviate substantially from the objective risk estimates, calculated using the INFLUENCE-nomogram. In this context, the added value of the nomogram exists in clinical practice, especially in decision-making regarding personalised post-treatment surveillance. A potential explanation for the higher estimates could also be that some HCPs included the risk for second primary tumours (SPs) or distant metastasis (DM) in their estimates instead of only the risk for LRRs. This pitfall is often observed in patients as well. Whereas the risk for SPs can impact personalisation of surveillance, DM is not actively looked for during surveillance, as early detection of DMs does not improve survival [2]. These research findings should serve as a wake-up call to educate HCPs and patients about the aim of surveillance (detection of LRRs and SPs), while informing them about the often minimal risks.

An update of the first INFLUENCE-nomogram has recently been performed leading to the CE certified INFLUENCE 2.0 model, which uses random forest models [11]. The 2.0 version also includes the risk for SPs, besides the risk for LRRs. Since the primary aim of surveillance is to detect both LRRs and SPs, the INFLUENCE-nomogram can now support decision-making about personalised surveillance better.

Recent studies have shown that shared decision-making about personalised surveillance is desirable [10, 12]. General overestimations of the risk for LRR might hinder objective patient information which is required for shared decision-making. The objective risk estimation can therefore be incorporated into a tool, such as a patient decision aid, to support the process of shared decision-making regarding surveillance. In the patient decision-aid, information on the aim and options for surveillance can be provided to put the risk information and need for personalised surveillance in context for the patient [12].

### Strengths and limitations

To our knowledge, this the first study to examine HCPs perceptions of risks for LRRs after breast cancer. This study also knows limitations. First, the limited number of respondent, and uneven spread of respondents over de groups of HCPs (specialties and hospitals) might limit the generalisability of the results. This study was performed in six teaching hospitals and one academic hospital which is a strength due to different approaches to follow-up but which also limits generalisability to other types of institutions such as general, or specialised hospitals. Second, we did not ask about risks for metastasis, due to the fact that early detection of DM is not an aim of surveillance, because it does not influence prognosis. Although this was stated on the survey/mentioned in the explanation, we cannot completely rule out that some HCPs might have perceived these elements as part of the risk for recurrences when making estimations. Thirdly, GPs were invited from one of the researchers’ networks. However, we do not feel that this has biased the results. Fourthly, we have no insight in the numbers of breast cancer patients that GPs see in their clinical practice. These numbers could vary and might have had an effect on the estimations of the GPs. Furthermore, we did not add the type of surgery (e.g. lumpectomy or mastectomy) and the HER-2 status in the patient cases. These factors are known to influence the risk for LRR, but could not be included in the cases because they were not yet included in the INFLUENCE-nomogram version 1.0. In future research, these factors can be included. Lastly, we currently have no insight in what influenced HCPs to estimate a certain risk for LRRs. For future studies, it would be interesting to also qualitatively explore which factors influence HCPs estimations of the risk for LRRs.

## Conclusions

Risks for LRRs estimated by HCPs deviate substantially from the objective risk estimates as calculated using the INFLUENCE-nomogram. Creating more awareness of the risk for LRRs is an important pre-requisite for the implementation of personalised surveillance after breast cancer. Besides education of HCPs, the use of prediction models such as the INFLUENCE-nomogram can support in estimating an objective estimate of each individual patient’s risk. The risk estimate calculated by the INFLUENCE-nomogram can also be combined with patient information, e.g. in a decision-aid, to support decision-making about personalised post-treatment surveillance.

## Data Availability

The datasets generated during and/or analysed during the current study are available from the corresponding author on reasonable request.

## Appendices

### Appendix I: Survey

#### Risk for locoregional recurrences within 5 years after breast cancer

- Medical oncologist
- Radiologist
- Nurse practitioner
- Breast cancer nurse
- Surgical oncologist
- Resident
- Radiotherapist
- General practitioner
- Other, namely: …

### Appendix II: Table risk estimations per case per discipline

**Table:**

	Discipline	Median	Mean	*N*	Std. deviation	Minimum	Maximum
Case 1A 0.96%^a^	Breast Cancer Nurse	10.0	11.25	4	6.29	5.0	20.0
	General Practitioner	5.0	6.16	25	4.23	1.5	20.0
	Medical Oncologist	2.0	2.00	5	0.71	1.0	3.0
	Nurse Practitioner	5.0	7.20	10	6.81	3.0	25.0
	Radiologist	4.0	6.89	9	9.48	0.0	30.0
	Radiotherapist	5.0	7.00	9	4.27	2.0	15.0
	Resident	2.0	3.35	13	3.29	0.5	10.0
	Surgical Oncologist	4.5	4.35	10	2.49	2.0	10.0
	All disciplines	5.0	5.80	85	5.30	0.0	30.0
Case 1B 2.29%^a^	Breast Cancer Nurse	11.0	11.75	4	2.36	10.0	15.0
	General Practitioner	10.0	13.30	25	9.60	4.0	40.0
	Medical Oncologist	3.0	3.40	5	1.67	2.0	6.0
	Nurse Practitioner	7.50	10.00	10	7.82	4.0	30.0
	Radiologist	10.0	16.78	9	21.96	0.0	70.0
	Radiotherapist	10.0	10.44	9	5.61	4.0	20.0
	Resident	7.0	7.12	13	4.62	0.5	15.0
	Surgical Oncologist	6.0	7.00	10	5.48	1.0	20.0
	All disciplines	8.0	10.64	85	10.07	0.0	70.0
Case 2 7.17^a^	Breast Cancer Nurse	20.0	22.50	4	5.00	20.0	30.0
	General Practitioner	18.0	18.56	25	10.15	5.0	45.0
	Medical Oncologist	6.0	7.00	5	2.83	4.0	10.0
	Nurse Practitioner	25.0	27.50	10	19.41	3.0	60.0
	Radiologist	15.0	19.78	9	14.55	5.0	50.0
	Radiotherapist	20.0	19.56	9	12.47	4.0	40.0
	Resident	10.0	11.31	13	7.81	3.0	25.0
	Surgical Oncologist	12.5	13.70	10	5.98	5.0	20.0
	All disciplines	15.0	17.67	85	12.16	3.0	60.0
Case 3A 1.18%^a^	Breast Cancer Nurse	7.50	11.25	4	9.46	5.0	25.0
	General Practitioner	5.00	8.96	25	6.89	1.0	30.0
	Medical Oncologist	3.00	2.90	5	1.75	0.5	5.0
	Nurse Practitioner	4.50	6.70	10	5.60	2.0	20.0
	Radiologist	5.00	10.67	9	12.25	0.0	30.0
	Radiotherapist	5.00	5.22	9	2.44	2.0	10.0
	Resident	3.00	4.65	13	5.27	0.5	20.0
	Surgical Oncologist	4.00	3.65	10	1.20	2.0	5.0
	All disciplines	5.00	6.95	85	6.80	0.0	30.0
Case 3B 2.27%^a^	Breast Cancer Nurse	12.50	12.50	4	2.89	10.0	15.0
	General Practitioner	15.00	15.24	25	8.34	5.0	40.0
	Medical Oncologist	8.00	6.6	5	3.97	1.0	10.0
	Nurse Practitioner	11.50	11.40	10	8.40	2.0	30.0
	Radiologist	15.00	21.01	9	23.76	0.1	70.0
	Radiotherapist	10.00	9.00	9	3.20	5.0	15.0
	Resident	8.00	9.66	13	7.37	0.6	30.0
	Surgical Oncologist	6.50	9.10	10	8.28	1.0	30.0
	All disciplines	10.00	12.53	85	10.70	0.1	70.0
Case 4A 0.74%^a^	Breast Cancer Nurse	12.50	12.50	4	2.89	10.0	15.0
	General Practitioner	15.00	15.88	25	7.38	2.0	40.0
	Medical Oncologist	5.00	4.60	5	0.89	3.0	5.0
	Nurse Practitioner	10.00	12.60	10	7.81	3.0	30.0
	Radiologist	10.00	13.67	9	15.29	1.0	50.0
	Radiotherapist	10.00	13.44	9	8.99	3.0	30.0
	Resident	8.00	9.15	13	5.83	3.0	20.0
	Surgical Oncologist	10.00	11.10	10	5.65	5.0	20.0
	All disciplines	10.00	12.59	85	8.37	1.0	50.0
Case 4B 1.72%^a^	Breast Cancer Nurse	20.00	17.50	4	8.66	5.0	25.0
	General Practitioner	25.00	25.24	25	10.32	4.0	60.0
	Medical Oncologist	6.00	6.80	5	1.92	5.0	10.0
	Nurse Practitioner	12.00	17.40	10	11.82	3.0	40.0
	Radiologist	15.00	20.72	9	23.61	1.5	80.0
	Radiotherapist	15.00	18.39	9	12.75	0.5	40.0
	Resident	10.00	15.62	13	10.89	2.0	35.0
	Surgical Oncologist	14.50	15.60	10	8.26	7.0	30.0
	All disciplines	19.00	19.06	85	12.86	0.5	80.0
Case 5A 1.57%^a^	Breast Cancer Nurse	20.00	21.25	4	6.29	15.0	30.0
	General Practitioner	25.00	25.68	25	11.60	5.0	50.0
	Medical Oncologist	6.00	5.80	5	1.92	3.0	8.0
	Nurse Practitioner	16.00	18.50	10	10.96	3.0	35.0
	Radiologist	15.00	20.94	9	18.89	1.5	60.0
	Radiotherapist	30.00	25.78	9	17.43	5.0	60.0
	Resident	10.00	17.69	13	14.21	4.0	50.0
	Surgical Oncologist	15.00	15.70	10	7.54	3.0	30.0
	All disciplines	20.00	20.57	85	13.38	1.5	60.0
Case 5B 3.6%^a^	Breast Cancer Nurse	30.00	26.25	4	11.09	10.0	35.0
	General Practitioner	30.00	36.14	25	15.23	10.0	70.0
	Medical Oncologist	10.00	11.00	5	2.65	8.0	15.0
	Nurse Practitioner	25.00	30.00	10	20.82	5.0	70.0
	Radiologist	20.00	30.28	9	25.87	2.5	90.0
	Radiotherapist	42.50	42.00	8	25.28	6.0	80.0
	Resident	20.00	23.54	13	16.60	4.0	60.0
	Surgical Oncologist	22.50	21.50	10	9.55	7.0	40.0
	All disciplines	25.00	29.68	84	18.70	2.5	90.0
Case 6A 0.64%^a^	Breast Cancer Nurse	12.50	12.50	2	10.61	5.0	20.0
	General Practitioner	8.00	9.26	25	5.73	2.0	20.0
	Medical Oncologist	1.00	1.25	4	0.50	1.0	2.0
	Nurse Practitioner	10.00	10.44	9	9.15	3.0	30.0
	Radiologist	2.50	2.50	2	3.54	0.0	5.0
	Radiotherapist	5.00	3.67	3	2.31	1.0	5.0
	Resident	5.00	4.56	9	3.00	1.0	10.0
	Surgical Oncologist	5.00	4.67	9	2.40	2.0	10.0
	All disciplines	5.00	7.21	63	6.11	0.0	30.0
Case 6B 2.94%^a^	Breast Cancer Nurse	14.00	14.00	2	8.49	8.0	20.0
	General Practitioner	12.00	15.48	25	8.18	5.0	30.0
	Medical Oncologist	2.00	2.25	4	1.26	1.0	4.0
	Nurse Practitioner	12.50	17.50	8	14.92	3.0	40.0
	Radiologist	5.05	5.05	2	7.00	0.1	10.0
	Radiotherapist	3.00	5.00	3	4.36	2.0	10.0
	Resident	7.00	8.28	9	5.38	0.5	18.0
	Surgical Oncologist	7.00	8.22	9	4.38	1.0	15.0
	All specialisms	10.00	11.90	62	9.18	0.1	40.0

^a^LRR estimates determined with the INFLUENCE-nomogram.

## References

[1] Integraal Kankercentrum Nederland (IKNL). NKR cijfers [NCR numbers]. https://iknl.nl/nkr-cijfers?fs%7Cepidemiologie_id=526&fs%7Ctumor_id=1&fs%7Cregio_id=550&fs%7Cperiode_id=564%2C565%2C566%2C567%2C568%2C569%2C570%2C571%2C572%2C573%2C574%2C575%2C576%2C577%2C578%2C579%2C580%2C581%2C582%2C583%2C584%2C585%2C586%2C587%2C588%2C589%2C590%2C591%2C592%2C593%2C563%2C562%2C561&fs%7Cgeslacht_id=644&fs%7Cleeftijdsgroep_id=677&fs%7Cjaren_na_diagnose_id=687&fs%7Ceenheid_id=703&cs%7Ctype=line&cs%7CxAxis=periode_id&cs%7Cseries=epidemiologie_id&ts%7CrowDimensions=periode_id&ts%7CcolumnDimensions=&lang%7Clanguage=en

[2] NABON (2012). Breast cancer - Dutch Guideline, version 2.0. Oncoline. https://www.oncoline.nl/uploaded/docs/mammacarcinoom/Dutch%20Breast%20Cancer%20Guideline%202012.pdf

[3] Geurts SME, de Vegt F, Siesling S (2012). Pattern of follow-up care and early relapse detection in breast cancer patients. Breast Cancer Res Treat.

[4] Geurts YM, Witteveen A, Bretveld R (2017). Patterns and predictors of first and subsequent recurrence in women with early breast cancer. Breast Cancer Res Treat.

[5] Witteveen A, Vliegen IM, Sonke GS (2015). Personalisation of breast cancer follow-up: a time-dependent prognostic nomogram for the estimation of annual risk of locoregional recurrence in early breast cancer patients. Breast Cancer Res Treat.

[6] Van der Sangen MJC, Scheepers SWM, Poortmans PMP (2012). Detection of local recurrence following breast-conserving treatment in young women with early breast cancer: optimization of long-term follow-up strategies. Breast.

[7] Cortazar P, Zhang L, Untch M, Mehta K, Costantino JP, Wolmark N (2014). Pathological complete response and long-term clinical benefit in breast cancer: the CTNeoBC pooled analysis. Lancet.

[8] Wishart GC, Bajdik CD, Dicks E, Provenzano E, Schmidt MK, Sherman M (2012). PREDICT Plus: development and validation of a prognostic model for early breast cancer that includes HER2. Br J Cancer.

[9] Zikmund-Fisher BJ, Janz NK, Hawley ST, Griffith KA, Sabolch A, Jagsi R (2016). Communication of recurrence risk estimates to patients diagnosed with breast cancer. JAMA Oncol.

[10] Ankersmid JW, van Hoeve JC, Strobbe LJA, van Riet YEA, van Uden-Kraan CF, Siesling S, Drossaert CHC, the Santeon VBHC Breast Cancer Group (2021). Follow-up after breast cancer: variations, best practices, and opportunities for improvement according to health care professionals. Eur J Cancer Care.

[11] Völkel V, Hueting TA, Draeger T, van Maaren MC, de Munck L, Strobbe LJA, Sonke GS, Schmidt MK, van Hezewijk M, Groothuis-Oudshoorn CGM, Siesling S (2021). Improved risk estimation of locoregional recurrence, secondary contralateral tumors and distant metastases in early breast cancer: the INFLUENCE 2.0 model. Breast Cancer Res Treat.

[12] Ankersmid JW, Drossaert CHC, van Riet YEA (2022). Needs and preferences of breast cancer survivors regarding outcome-based shared decision-making about personalised post-treatment surveillance. J Cancer Surviv.

